# Re: Book review: O.S. Miettinen: Up from clinical epidemiology & EBM

**DOI:** 10.1007/s10654-012-9690-x

**Published:** 2012-05-11

**Authors:** Jan Van den Broeck, Jonathan R. Brestoff

**Affiliations:** 1 Centre for International Health, University of Bergen, Bergen, Norway; 2 Perelman School of Medicine, University of Pennsylvania, Philadelphia, PA USA

In a logical sequence of progressively deduced propositions and principles, Dr. Miettinen attempts to re-orient medical academia to the theories underlying clinical research in his recent book *Up from Clinical Epidemiology & EBM* (Evidence Based Medicine) [1]. This ambitious avant-garde text first examines the role of medical academia and criticizes—perhaps too harshly at times—the current ways of achieving knowledge about diagnosis, etiognosis, and prognosis in clinical medicine. Currently lacking for clinicians is an immediately applicable scientific knowledge base of clinical practice. Instead, in the current clinical epidemiology culture, practitioners are advised to spend lengths of time critically appraising literature on particular topics, form their own opinions on it, and use their uncodified experiences and varying opinions to make clinical decisions. The aim of EBM to standardize clinical practice is stymied by the process currently required to employ EBM. Exacerbating the problem are widespread imperfections in designs of clinical studies. For these reasons and others, Miettinen argues that EBM is a fallacy, “a cult movement…at variance with the essence of science and the imperatives of professionalism in medicine.”

In place of current EBM, Miettinen suggests different approaches to diagnosis, etiognosis, and prognosis—ones that are arguably more valid and objective and that may have the potential to bring major improvements to clinical medicine. To construct a proper knowledge base for clinical practice and offer it in a more directly usable form, Miettinen proposes the construction and use of ‘gnostic probability functions.’ These address the probability of an illness being present, a risk factor having played a causal role, or an untoward event happening in the course of an illness as a function of diagnostic, etiognostic, or prognostic indicators, respectively.

Miettinen develops in his book many new ideas about diagnosis, so we focus our review mainly to these. In support of diagnosis, Miettinen proposes that diagnostic probability functions (DPF) be used instead of ‘reverse probabilities’ (i.e., probabilities derived from comparing past signs and symptoms in series of patients and non-patients) and Bayes factors now commonly used in clinical epidemiology. Miettinen describes a strategy of constructing DPF using codified tacit knowledge of experts. We agree with Lubsen [2] that the description of this strategy is one of the major contributions of the book. Basically, panels of experts are presented with hypothetical patient presentations and asked to attach a probability to each scenario that a particular illness is present. Using the responses from many experts, multiple logistic regression analysis is then employed to model the probability of an illness as a function of diagnostic indicators, which include components of the risk profile (e.g., socio-demographic factors) and manifestation profile (e.g., symptoms, signs, and test results). The resulting model is incorporated into software that allows users to estimate diagnostic probabilities for real presenting patients. A derived method provides for the evaluation of diagnostic tests as well. As Miettinen states, “In this Information Age, the implication is that the availability of user-friendly gnostic expert systems would enhance the efficiency of healthcare by inherently contributing to both quality assurance and cost containment in it.”

Assuming that valid DPF are defined for a wide enough range of clinical scenarios, we too see the approach of codifying experts’ tacit knowledge and the e-implementation of DPF as having great promise in supporting clinical diagnosis, especially in settings where clinical expertise is limited or inaccessible. However, a probability function will be only as good as the data used to fit such a model. We note that those data will be limited by the ability of experts to recognize and accurately describe all aspects of an illness process or presentation, and must be regularly updated to accommodate evolving perspectives of disease and continuous biomedical innovation. There may be a role here for cloud-based artificial intelligence.

The scale of research efforts needed to construct such detailed descriptions of diagnostic profiles and the challenges of implementing DPF seem so daunting that we are sceptical about the practical nature of this great idea. Moreover, it is unclear to us how well such models can incorporate local effect-modifying circumstances as part of the risk profile, or how useful they will be for atypical clinical presentations. Yet even if the use of DPF is not feasible for making refined diagnoses, we wonder about the value of their application for the specific purpose of triage.

A major theme in *Up from Clinical Epidemiology & EBM* is that several currently popular general study designs—examples of which include the cohort, case–control, and traditional diagnostic performance studies—are fallacies. This theme has also dominated many of Miettinen’s previous writings. We agree with him that classical cohort studies fail to see etiologic time as inherently negative; that classical case–control approaches fail to define the reference series from the study base; and that traditional diagnostic studies are limited by their reverse orientation and inability to clearly define a study domain. For Miettinen these imperfections, together with his view that particularistic studies are not scientific and not even research, seem to imply the need for a tabula rasa approach, a blank slate. Consequently, the range of general study designs considered in this book is narrower than what many readers will expect.

Readers should be advised that reading this book is a serious undertaking, and we recommend using Miettinen’s other recent book *Epidemiological Research: Terms and Concepts* [3–5] in support. By questioning widely held assumptions and using logical deductive reasoning, Miettinen invokes his Socratic nature and confirms his great strength as a theoretical epidemiologist. We highly recommend this fascinating book to all epidemiologists and look forward to the much-needed discourse on its content.
